# Clinical application of Myxovirus resistance protein A as a diagnostic biomarker to differentiate viral and bacterial respiratory infections in pediatric patients

**DOI:** 10.3389/fimmu.2025.1540675

**Published:** 2025-02-19

**Authors:** Min Zhu, Lijin Chen, Jiali Cao, Jianguo Cai, Shuying Huang, Huibin Wang, Huanjuan He, Zimin Chen, Rongfu Huang, Huiming Ye

**Affiliations:** ^1^ Department of Laboratory Medicine, Department of Pediatrics, Fujian Key Clinical Specialty of Laboratory Medicine, Women and Children’s Hospital, School of Medicine, Xiamen University, Xiamen, China; ^2^ Department of Pathology, Xiamen Medical College Affiliated Haicang Hospital, Xiamen, China; ^3^ Department of Prevention and Treatment of Endemic and Chronic Diseases, Disease Prevention and Control Center of Xiuyu District, Putian, China; ^4^ Department of Research and Development, Xiamen Innobiomax Biotechnology Co., Ltd, Xiamen, China; ^5^ Department of Clinical Laboratory, The Second Affiliated Hospital, Fujian Medical University, Quanzhou, China

**Keywords:** Myxovirus resistance protein A (MxA), pediatric respiratory infections, biomarker, bacterial versus viral infection, diagnostic accuracy

## Abstract

**Background:**

Differentiating between viral and bacterial respiratory tract infections in pediatric patients remains a significant diagnostic challenge, often leading to the overuse of antibiotics. Myxovirus resistance protein A (MxA) has been identified as a promising biomarker for viral infections. This study aimed to assess the fluctuations in blood MxA levels among children with viral respiratory infections and to explore the differences in MxA levels between viral and bacterial infections, focusing on clinical implications for antibiotic use.

**Methods:**

We conducted a retrospective study using enzyme-linked immunosorbent assay (ELISA) to measure MxA levels in a cohort of 314 children with respiratory tract infections and 89 healthy controls. The study compared MxA levels across children with viral, bacterial, and mixed infections. Diagnostic accuracy was evaluated using receiver operating characteristic (ROC) curve analysis to distinguish between viral and bacterial infections or between viral and co-infections, with additional comparisons to other established infection biomarkers.

**Results:**

MxA levels were significantly elevated in children with viral infections (n=205) compared to bacterial infections (n=21) (p<0.0001). The ROC curve analysis demonstrated that MxA had an area under the curve (AUC) of 0.8019 (95% CI: 0.6989 to 0.9049) for distinguishing viral from bacterial infections. Combining MxA with C-reactive protein (CRP) further enhanced diagnostic performance, achieving an AUC of 0.8713 (95% CI: 0.7916 to 0.9510). However, the use of MxA or MxA/CRP alone is insufficient to differentiate viral and viral - bacterial coinfection. The AUC of MxA is 0.5161 (95% CI: 0.4392 to 0.5930), and the AUC of MxA/CRP is 0.5429 (95% CI: 0.4705 to 0.6153).

**Conclusions:**

This study highlights the diagnostic potential of MxA as a biomarker for differentiating viral from bacterial respiratory infections in children. The combined use of MxA and CRP offers a novel approach to improve diagnostic accuracy. Still, a combination with other clinical and laboratory markers remains required to determine whether to administer antibiotics to children with respiratory tract infections.

## Introduction

Respiratory infections remain a significant global threat to child health, contributing to a substantial proportion of pediatric mortality (1–3). Respiratory infections in early childhood have a significant impact on the broncho-alveolar and vascular development of the lungs which cause long-term effects on the health of children (4). Viruses and bacteria are the primary causative agents of these infections, making the accurate identification of pathogens essential for effective treatment (5).

Currently, the differentiation between viral and bacterial respiratory infections in pediatric patients relies largely on clinical symptoms, leukocyte count and classification, erythrocyte sedimentation rate (ESR), C-reactive protein (CRP), and other laboratory indicators (6–8). However, these diagnostic tools lack specificity and often show limited sensitivity, complicating the accurate diagnosis of the infection type. While current biomarkers can estimate the likelihood of bacterial infections, there remains a critical gap in reliable biomarkers for detecting viral infections. This diagnostic gap underscores the urgent need for novel biomarkers, especially given the strict antibiotic stewardship regulations in many countries that necessitate accurate differential diagnoses to avoid unnecessary antibiotic prescriptions (9, 10).

Recent advancements in basic research have led to the discovery and development of novel biomarkers for infectious diseases. Notably, in the context of respiratory viral infections, the host immune response triggers the production of interferons, which subsequently induce the synthesis of Myxovirus resistance protein A (MxA). MxA, primarily activated by type I interferons (IFN-α/β), has gained significant attention due to its potential as a diagnostic marker for viral infections (11, 12). Studies have demonstrated that MxA holds diagnostic value in distinguishing viral infections from bacterial infections in both pediatric and adult populations. Furthermore, integrating MxA with specific bacterial infection markers has been explored to improve the management of children presenting with respiratory infection symptoms (13, 14). Despite these promising findings, more comprehensive research is needed to fully leverage the diagnostic potential of MxA and other emerging biomarkers in clinical practice (15).

This study aims to provide a systematic evaluation of blood MxA levels among children with viral, bacterial, and mixed respiratory infections compared to healthy controls in Xiamen, a coastal city in southeastern China. The findings highlight the diagnostic potential of MxA for distinguishing viral from bacterial infections. Additionally, we propose a novel diagnostic strategy that combines MxA with CRP to enhance the accuracy of differentiating between viral and bacterial respiratory infections in clinical settings in China.

## Materials and methods

### Study design and classification

This study is a retrospective analysis. The enrollment period for all participants was from May 2023 to October 2023. This clinical investigation recruited a cohort of 314 children (aged 1 month to 15 years) diagnosed with respiratory tract infections, along with 89 age-matched healthy controls, at Xiamen Maternal and Child Health Hospital, Xiamen, China. Blood and nasopharyngeal aspirates were collected for biomarker analysis.

The viral infection group was defined by: (i) a clinical diagnosis of respiratory tract infection, (ii) absence of bacterial etiology, and (iii) detection of viral markers via multiplex PCR. The bacterial infection group was characterized by: (i) a clinical diagnosis of respiratory tract infection, (ii) identification of pathogenic bacteria from blood or nasopharyngeal aspirates (8/21, 38.10%), or clinical improvement with antibiotic treatment (13/21, 61.90%), and (iii) no clinical or microbiological evidence of viral infection. The effectiveness of antibiotic treatment is assessed three days after standardized therapy by two doctors, based on the following criteria: (i) clinical symptoms and signs, including a significant reduction in body temperature within 48–72 hours, marked alleviation of symptoms such as cough and sputum production, and stabilization of clinical status without further deterioration; (ii) laboratory tests, with a gradual decrease or normalization of total white blood cell count and a decline in inflammatory markers such as C-reactive protein (CRP) and procalcitonin (PCT); and (iii) imaging, if necessary, showing reduced or significantly absorbed lesions on follow-up chest X-rays or CT scans. The co-infection group included cases with: (i) a clinical diagnosis of respiratory tract infection, (ii) simultaneous detection of viral infection and pathogenic bacteria in blood or nasopharyngeal aspirates. The healthy control group criteria included: absence of respiratory tract infection, no use of antibacterial or antiviral medications within the past week, no chronic conditions (e.g., diabetes, hypertension), no major liver or kidney diseases, no coagulation disorders or active bleeding, and no autoimmune or neoplastic diseases. Healthy controls were children who visited the hospital for routine health examinations and met the inclusion criteria for the control group.

### Sample and measurement

Blood samples were obtained via venous puncture from both pediatric patients with illness and those who were healthy. The samples underwent various analyses, including blood bacterial culture, white blood cell (WBC) count and platelet count, determinations of C-reactive protein (CRP), Serum procalcitonin (PCT), interleukin-6 (IL-6) and amyloid A (SAA) levels using standard procedures in the hospital central laboratories. Subsequently, blood samples were stored at -70°C and then underwent unified measurement of MXA. The complete blood count reagent (resistance measurement method and flow cytometry) and CRP diagnostic reagent (immunoturbidimetry) are produced by Shenzhen Mindray Bio-Medical Electronics Co.,Ltd. Additionally, the whole blood MxA diagnostic reagent (ELISA), IL-6 (Chemiluminescence Microparticle Immunoassay, CMIA), SAA (CMIA), and PCT (CMIA), are produced by Xiamen InnoDx Biotech Co., Ltd., China. The measurement process of CMIA was conducted with automatic analyzer Caris200 (Xiamen UMIC Medical Instrument Co. Ltd., China).

The virus in nasopharyngeal aspirates was detected using SureX^®^ 13 Respiratory Pathogen Multiplex Detection Kit (Health Gene Technology, Ningbo, China), including influenza A virus, influenza B virus, adenovirus, bocavirus, rhinovirus, parainfluenza virus, coronavirus, respiratory syncytial virus, metapneumovirus, mycoplasma pneumoniae and chlamydia. The bacteria were detected using the classical culture method. The identification of bacterial strains was carried out utilizing time-of-flight mass spectrometer (microTyper MS, Zhongyuan Huiji, China) and a fully automated bacterial identification/drug sensitivity analysis system (Phoenix M50, BD Medical, USA).

### Statistical analysis

The Mann-Whitney U-test and Kruskal-Wallis nonparametric tests were used for the comparisons of MxA, CRP, PCT, IL-6 and SAA protein levels between groups as appropriate. ROC analysis was utilized to assess the discriminatory ability of blood indicator levels and blood MxA/CRP ratio between patient groups. Cutoff levels were calculated from the ROC analyses using the Youden index.

This study was approved by the Ethics Committee of Xiamen Maternal and Child Health Hospital (KY-2022-005-H01) and was performed following the Helsinki Declaration of 1964 and its later amendments. Medical records were deidentified for all personally identifiable information.

## Result

This study enrolled a total of 314 pediatric patients diagnosed with respiratory tract infections, alongside a healthy control group consisting of 89 children. Among the 314 patients, 205 were diagnosed with viral infections, 21 with bacterial infections, and 88 with mixed infections involving both viral and bacterial pathogens. Detailed clinical characteristics and specific diagnoses for these patient groups are summarized in **Table 1**. Notably, the most frequently observed conditions were acute upper respiratory tract infections and bronchopneumonia.

**Table 1:** Clinical characteristics and diagnoses of pediatric patients.

Characteristic	HC	Viral infection	Bacterial infection	Co-infection
Sample size	89	205	21	88
Age				
1-11 mo	11 (12.36%)	84 (40.98%)	9 (42.86%)	44 (50.00%)
1-2 yr	8 (8.99%)	64 (31.22%)	6 (28.57%)	25 (28.41%)
3-6 yr	28 (31.46%)	55 (26.83%)	5 (23.81%)	18 (20.45%)
7-15 yr	42 (47.19%)	2 (0.98%)	1 (4.76%)	1 (1.14%)
Sex				
Female	39	83	7	23
Male	50	122	14	65
Clinical diagnoses				
Acute upper respiratory tract infection	–	38	6	3
Herpetic pharyngitis	–	6	1	0
Tonsillitis	–	6	2	0
Bronchopneumonia	–	102	9	58
Severe pneumonia	–	29	1	16
Bronchitis	–	31	2	9
Pneumonia	–	3	0	1
Laryngitis	–	0	0	1

### MxA as a biomarker for diagnosing pediatric respiratory viral infections

**Figure 1:**
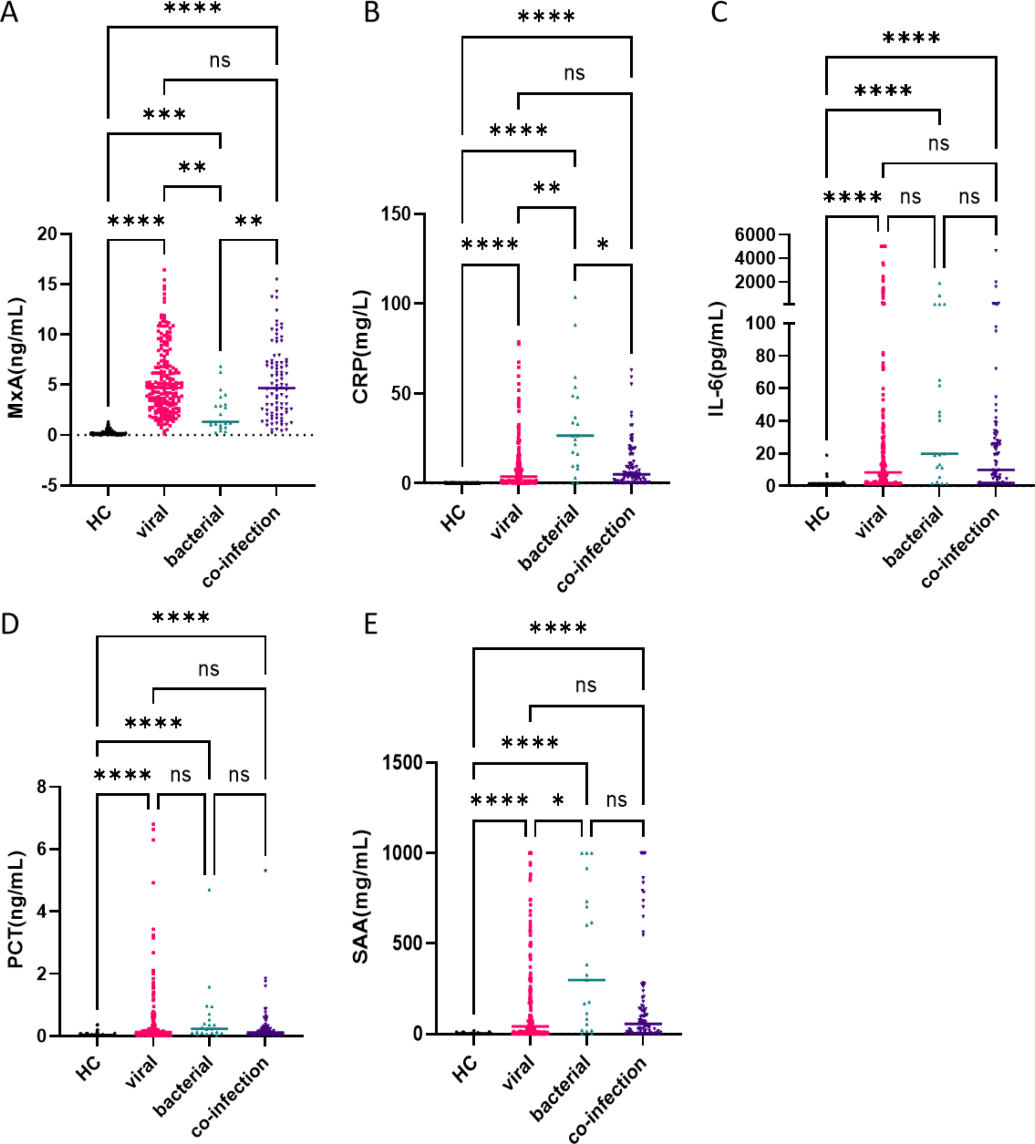
Biomarker levels in blood samples from children with respiratory tract bacterial infection, viral infection, co-infection and healthy controls. The level of **(A)** MxA protein, **(B)** PCT protein, **(C)** CRP protein, **(D)** IL-6 protein and **(E)** SAA protein. For each group, the horizontal line represents the median. For pairwise comparisons of the groups “viral infection” and “viral-bacterial coinfection” with “bacterial infection” and “healthy control (HC)” for all comparisons as determined by the Mann-Whitney U test. ****p<0.0001; ***p<0.001; **p<0.01; *p<0.05; ns, no significant difference.

The analysis of blood samples indicated that MxA levels were significantly elevated in both the viral infection and co-infection groups when compared to the healthy control group (P < 0.0001) (**Figure 1A**). No significant difference was found between MxA levels in the viral infection group and those in the co-infection group. The MxA levels in the bacterial infection group were significantly lower than in the viral infection group (P < 0.01). Interestingly, the bacterial infection group also displayed significantly higher MxA levels compared to the healthy controls (P < 0.001).

Biomarkers, including C-reactive protein (CRP), procalcitonin (PCT), and interleukin-6 (IL-6), in the bacterial infection group exhibited significantly elevated levels relative to the healthy controls (P < 0.0001) (**Figures 1B–D**). These markers were markedly lower in the viral infection group compared to the bacterial infection group, with CRP showing the most significant difference. Additionally, serum amyloid A (SAA), another key infection biomarker, was found to be significantly elevated in both the bacterial and co-infection groups compared to the healthy controls. However, SAA levels were significantly lower in the viral infection group than in the bacterial infection group (P < 0.05) (**Figure 1E**).

### Diagnostic performance of MxA and inflammatory biomarkers in differentiating viral from bacterial respiratory infections

**Figure 2:**
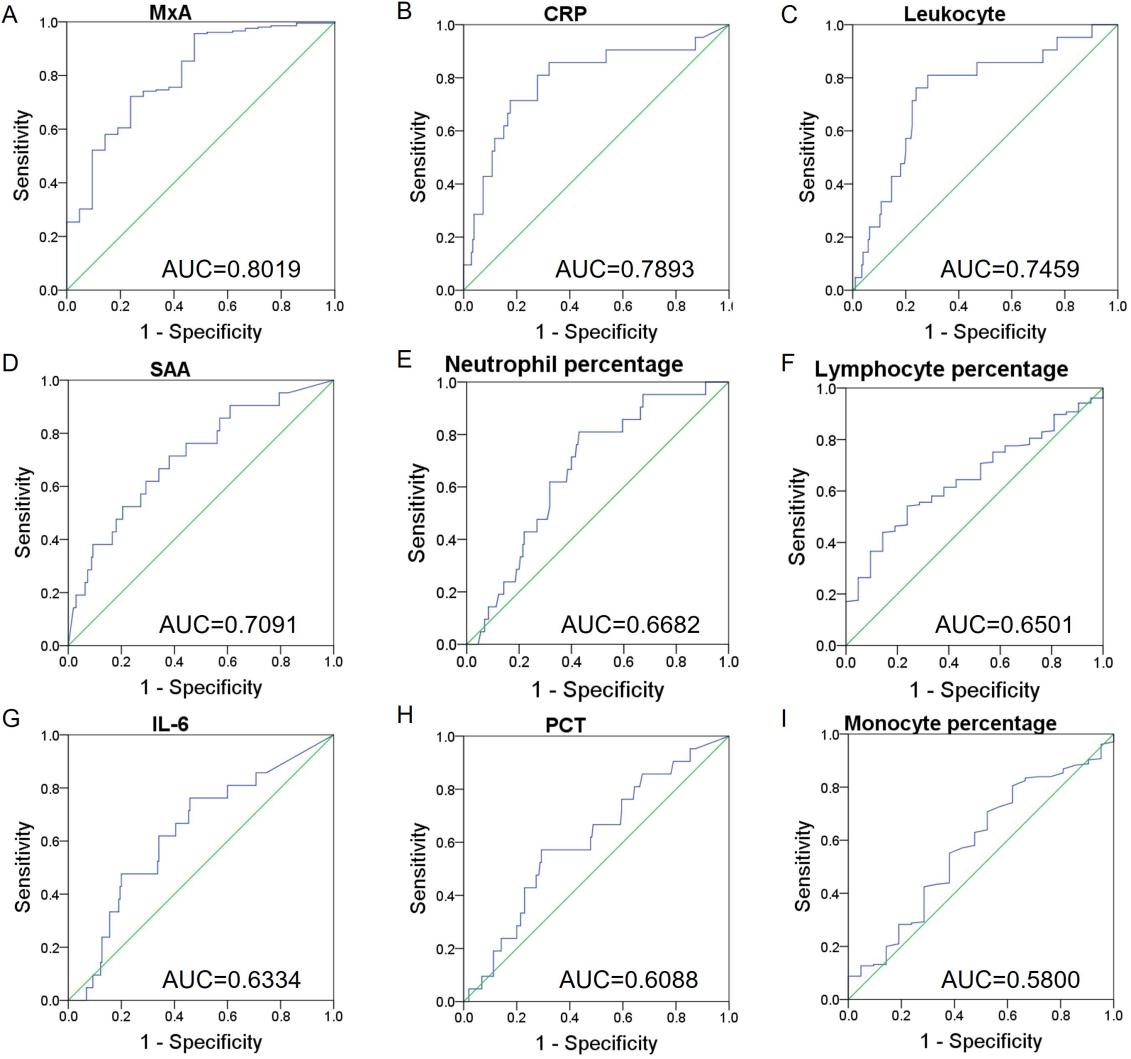
Differentiation between viral and bacterial infections by indicators. ROC curves for **(A)** MxA protein levels, **(B)** CRP protein levels, **(C)** leukocyte counts, **(D)** SAA protein levels, **(E)** neutrophils percentages, **(F)** lymphocyte percentages, **(G)** IL-6 protein levels, **(H)** PCT protein levels, and **(F)** monocyte percentages in blood samples differentiating between children with viral or bacterial infection.

The diagnostic performance of MxA and other inflammatory biomarkers in distinguishing viral from bacterial respiratory infections was evaluated using ROC curve analysis. Among all the biomarkers analyzed, MxA exhibited the highest diagnostic accuracy, with an AUC of 0.8019 (95% CI: 0.6989–0.9049). CRP and Leukocyte count also demonstrated strong diagnostic utility, with AUCs of 0.7893 (95% CI: 0.6717–0.9069) and 0.7459 (95% CI: 0.6327–0.8590), respectively. SAA showed moderate performance with an AUC of 0.7091 (95% CI: 0.5889–0.8292), while neutrophil percentage, lymphocyte percentage, IL6, PCT and monocyte percentage yielded lower AUCs (**Table 2**; **Figure 2**).

**Table 2:** Performance characteristics for ROC curve for differentiation between viral and bacterial infections.

Indicator	AUC	95%CI	Cutoff
MxA	0.8019	0.6989 to 0.9049	3.08
CRP	0.7893	0.6717 to 0.9069	16.13
Leukocyte	0.7459	0.6327 to 0.8590	10.84
SAA	0.7091	0.5889 to 0.8292	81.07
Neutrophil percentage	0.6682	0.5622 to 0.7741	47.05
Lymphocyte percentage	0.6501	0.5457 to 0.7544	40.30
IL6	0.6334	0.5104 to 0.7565	10.97
PCT	0.6088	0.4842 to 0.7335	0.22
Monocyte percentage	0.5800	0.4478 to 0.7122	7.05

### The MxA/CRP ratio as a diagnostic biomarker for differentiating viral and bacterial respiratory infections

**Figure 3:**
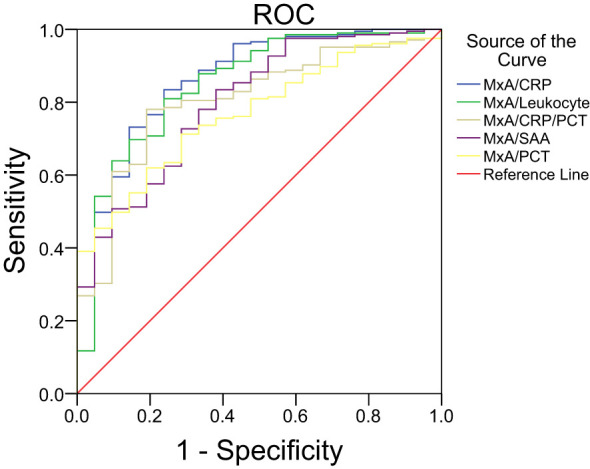
Differentiation between viral and bacterial infections by MxA/CRP ratio and other composite biomarkers.

The performance of MxA/CRP and other composite biomarkers in differentiating viral from bacterial respiratory infections was evaluated (**Table 3**; **Figure 3**). The MxA/CRP ratio demonstrated the highest diagnostic accuracy among the composite biomarkers, with an AUC of 0.8713 (95% CI: 0.7916–0.9510). The MxA/Leukocyte ratio also showed strong diagnostic utility, achieving an AUC of 0.8497 (95% CI: 0.7545–0.9449). Other composite biomarkers, including MxA/CRP/PCT and MxA/SAA, exhibited moderate diagnostic performance, with AUCs of 0.8037 (95% CI: 0.7114–0.8961) and 0.8005 (95% CI: 0.7012–0.8997), respectively. The MxA/PCT ratio had comparatively lower diagnostic accuracy, with an AUC of 0.7682 (95% CI: 0.6810–0.8554).

**Table 3:** Performance characteristics of composite biomarkers for ROC curve for differentiation between viral and bacterial infections.

Indicator	AUC	95%CI	Cutoff
MxA/CRP	0.8713	0.7916 to 0.9510	0.20
MxA/Leukocyte	0.8497	0.7545 to 0.9449	0.27
MxA/CRP/PCT	0.8037	0.7114 to 0.8961	0.83
MxA/SAA	0.8005	0.70123 to 0.8997	0.01
MxA/PCT	0.7682	0.6810 to 0.8554	20.25

### Diagnostic performance of biomarkers in differentiating viral infections from mixed infections

The ROC analysis for distinguishing viral infections from mixed infections revealed that MxA and its combinations with CRP or other markers had poor diagnostic performance (**Table 4**). MxA alone yielded an AUC of only 0.5161 (95% CI: 0.4392–0.5930), indicating limited utility. Similarly, composite biomarkers such as MxA/CRP and MxA/CRP/PCT also showed weak performance, with AUCs of 0.5429 (95% CI: 0.4705–0.6153) and 0.5225 (95% CI: 0.4517–0.5933), respectively. Other ratios, including MxA/SAA and MxA/Leukocyte, were also suboptimal, with AUCs of 0.5342 (95% CI: 0.4629–0.6055) and 0.5699 (95% CI: 0.4968–0.6430), further demonstrating the challenges of using these markers to differentiate viral from mixed infections. Among all the tested biomarkers, leukocyte count exhibited the relatively best performance, with an AUC of 0.6179 (95% CI: 0.5502–0.6856), though its diagnostic accuracy was still moderate.

**Table 4:** Performance characteristics of different indicators for ROC curve for differentiation between viral and co-infections.

Indicator	AUC	95%CI	Cutoff
Leukocyte	0.6179	0.5502 to 0.6856	8.04
MxA/Leukocyte	0.5699	0.4968 to 0.6430	0.51
MxA/CRP	0.5429	0.4705 to 0.6153	2.28
Monocyte percentage	0.5350	0.4617 to 0.6083	6.35
PCT	0.5343	0.4623 to 0.6063	0.09
MxA/SAA	0.5342	0.4629 to 0.6055	1.06
CRP	0.5270	0.4552 to 0.5987	1.50
SAA	0.5230	0.4521 to 0.5940	16.99
MxA/CRP/PCT	0.5225	0.45169 to 0.5933	5.22
MxA	0.5161	0.4392 to 0.5930	5.89
Lymphocyte percentage	0.5157	0.4438 to 0.5875	57.85
Neutrophil percentage	0.5107	0.43793 to 0.5834	42.30
IL6	0.5105	0.4388 to 0.5822	18.80
MxA/PCT	0.5062	0.4366 to 0.5757	32.14

## Discussion

In this study, we demonstrated that MxA has good diagnostic performance in distinguishing viral from bacterial respiratory tract infections in pediatric patients, with an AUC of 0.8019 (95% CI: 0.6989–0.9049). Furthermore, combining MxA with CRP further enhanced the diagnostic accuracy, as the MxA/CRP ratio achieved an AUC of 0.8713 (95% CI: 0.7916 to 0.9510), highlighting its potential as a powerful diagnostic tool in this context. However, both MxA alone and the MxA/CRP ratio showed poor performance in differentiating viral infections from mixed infections, with AUCs of 0.5161 and 0.5429, respectively. These findings suggest that while MxA and its combination with CRP are effective for distinguishing viral and bacterial infections, they are insufficient for mixed infections. To address this limitation, integrating additional biomarkers or clinical indicators may be necessary to improve the diagnostic accuracy for mixed infections.

Pediatric patients are particularly susceptible to respiratory tract infections due to the vulnerability of their developing respiratory systems. Without timely and accurate diagnosis, infections can progress to severe conditions like pneumonia, posing significant health risks (16). Currently, the early clinical differentiation between viral and bacterial infections largely relies on symptomatology, WBC counts, and markers like CRP and PCT (17, 18). Upon diagnosis of a bacterial infection, antibiotics are usually prescribed. To date, viral infections can be determined by molecular methods, which are expensive, time-consuming and complicated. Clinically, children with viral infections often present with a range of symptoms, such as respiratory symptoms, rash, fever, and meningitis. Once such suspicious clinical symptoms appear, antibiotics are frequently administered as a precautionary measure during the diagnostic process, leading to the overutilization of antibiotics (9, 19). Up to now, there has been a lack of commercially available immunoassay reagents capable of accurately distinguishing between various viral infectious diseases.

MxA is one of the antiviral proteins, distributed in the cytoplasm, which exhibits a broad spectrum of antiviral activities and exerts inhibitory effects on numerous RNA viruses and select DNA viruses, including influenza virus, enterovirus, and respiratory syncytial virus., etc (20, 21). Studies have shown that even a minimal viral load can stimulate the production of MxA protein in cells, whereas cells infected with bacteria, parasites, and other microorganisms do not exhibit induction of MxA protein expression (22–24). Therefore, the MxA protein expression levels in cells have been proposed as a potential indicator of viral infection, facilitating differential diagnosis from other microbial infections, notably bacterial (25). In recent years, several studies have demonstrated the potential diagnostic value of MxA protein levels in identifying viral infections; however, its integration into routine clinical practice remains limited (26, 27). This study provides new evidence to support the utilization of MxA as a diagnostic indicator for respiratory viral infections in clinical settings, and the combination of CRP with MxA improves the diagnostic value of MxA.

However, it is noteworthy that our study found slightly elevated MxA levels in the bacterial infection group compared to the control group. This observation may be due to challenges in differentiating between bacterial infections and viral-bacterial co-infections, as some bacterial cases may involve undetected viral components, leading to misclassification as purely bacterial infections. It is already commonly known that CRP is less specific than PCT in bacterial infections. CRP is an acute-phase protein produced by the liver in response to increases in proinflammatory cytokines such as IL-1β and IL-6, while PCT is produced by various cell types in response to bacterial cell wall components such as lipopolysaccharide, peptidoglycan, and lipoteichoic acid, as well as proinflammatory cytokines like IL-1β, IL-6, and TNF-α. However, in our study, CRP outperformed PCT in distinguishing viral from bacterial infections (AUC: 0.7893 vs. 0.6088). Similarly, another study has also demonstrated superior performance of CRP over PCT for this purpose (28). We hypothesize that this finding may be due to differences in the patient cohort or the underlying inflammatory responses in pediatric respiratory infections. Furthermore, viral-bacterial co-infections might modulate the levels of these biomarkers differently, potentially affecting their relative diagnostic utility. We also acknowledge that the relatively small sample size in our study might have influenced the results, and we emphasize the need for further research to validate these findings.

Theoretically, CRP is expected to perform well in distinguishing between viral infections and viral-bacterial co-infections. However, the results of this study revealed that CRP had limited effectiveness in differentiating between viral infections and viral-bacterial co-infections, while showing better performance in distinguishing bacterial infections from bacterial-viral co-infections. We hypothesize that this phenomenon may be attributed to several factors. In the viral-bacterial co-infection group, although bacteria were detected, some of the bacteria might have been colonizing rather than serving as primary pathogens, as colonizing bacteria typically do not significantly induce elevated CRP levels in the host. Additionally, viral infections might interfere with the CRP levels induced by bacterial infections through immune system regulation. Furthermore, the relatively small sample size in the bacterial infection group may have affected the robustness of the statistical analysis. The reasons also contribute to the inability of MxA/CRP to effectively distinguish between the viral infection group and the viral-bacterial co-infection group. In this study, MxA and the combined use of MxA/CRP can effectively differentiate viral infections from bacterial infections, but they fail to show significant differences between viral infections and mixed infections. This suggests that when MxA or MxA/CRP levels are elevated, it is not sufficient to distinguish between isolated viral infections and mixed infections. Therefore, decisions on antibiotic use cannot rely solely on MxA or MxA/CRP levels but must integrate other clinical markers, such as white blood cell count (WBC), C-reactive protein (CRP), and procalcitonin (PCT), as well as patient history and clinical symptoms.

The cohort of this study was relatively small, comprising 314 children with respiratory infections and 89 healthy controls, which may limit the generalizability of our findings to all respiratory infections. Additionally, all participants were recruited from Xiamen Maternal and Child Health Hospital, introducing potential regional and selection biases. In this study, the majority of patients in the infection group were children under 2 years of age, while the healthy control group consisted primarily of children aged 3-15 years. This discrepancy is a limitation of our retrospective analysis. It is primarily due to the fact that respiratory infections are more common in children under 2 years, whereas younger children undergoing routine health examinations rarely have blood samples collected, or only very small amounts of peripheral blood are obtained. As a result, it was challenging to recruit enough age-matched healthy controls for this study. Future studies should aim to expand the sample size, optimize the criteria for infection grouping, and incorporate MxA and other biomarkers to enhance the diagnostic accuracy for viral and bacterial infections.

To date, limited research has been conducted on the utilization of blood MxA protein level for the differential diagnosis of respiratory tract infections in Chinese pediatric populations. By using an enzymatic immunoassay to measure MxA, this study aims to promote more rational and targeted clinical management of pediatric respiratory infections, ultimately reducing unnecessary antibiotic use.

## Data Availability

The raw data supporting the conclusions of this article will be made available by the authors, without undue reservation.
